# HLA Expression Correlates to the Risk of Immune Checkpoint Inhibitor-Induced Pneumonitis

**DOI:** 10.3390/cells9091964

**Published:** 2020-08-25

**Authors:** Pierpaolo Correale, Rita Emilena Saladino, Diana Giannarelli, Andrea Sergi, Maria Antonietta Mazzei, Giovanna Bianco, Rocco Giannicola, Eleonora Iuliano, Iris Maria Forte, Natale Daniele Calandruccio, Antonia Consuelo Falzea, Alessandra Strangio, Valerio Nardone, Pierpaolo Pastina, Paolo Tini, Amalia Luce, Michele Caraglia, Daniele Caracciolo, Luciano Mutti, Pierfrancesco Tassone, Luigi Pirtoli, Antonio Giordano, Pierosandro Tagliaferri

**Affiliations:** 1 Medical Oncology Unit, Grand Metropolitan Hospital “Bianchi-Melacrino-Morelli”, 89124 Reggio Calabria, Italy (OU-RC); correalep@yahoo.it (P.C.); bianco.giovanna_@libero.it (G.B.); roccogiannicola@gmail.com (R.G.), eleonorafiuliano@hotmail.it (E.I.); danielecala@hotmail.com (N.D.C.); antonellafalzea@gmail.com (A.C.F.); alestr87@gmail.com (A.S.); 2 Tissue Typing Unit, Grand Metropolitan Hospital “Bianchi-Melacrino-Morelli”, 89124 Reggio Calabria, Italy (OU-RC); ritaemilena.saladino@gmail.com; 3 Biostatistical Unit, National Cancer Institute “Regina Elena”, IRCCS, 00161 Rome, Italy; diana.giannarelli@ifo.gov.it; 4 Radiology Unit, Grand Metropolitan Hospital “Bianchi-Melacrino-Morelli”, 89124 Reggio Calabria, Italy (OU-RC); andreasergi82@icloud.com; 5 Department of Medical, Surgical and Neuro-Sciences, Diagnostic Imaging, University of Siena, Azienda Ospedaliera Universitaria Senese, 53100 Siena, Italy (RU-SI); mamazzei@gmail.com; 6 Cell Biology and Biotherapy Unit, Istituto Nazionale Tumori-IRCCS-Fondazione G. Pascale, 80131 Naples, Italy; irisforte@yahoo.it; 7 Radiotherapy Unit, “Ospedale del Mare”, ASL Napoli 1, 80147 Naples, Italy; v.nardone@hotmail.it; 8 Section of Radiation Oncology, Medical School, University of Siena, 53100 Siena, Italy (ROU-SI); pastina.pierpaolo85@gmail.com (P.P.); paolo-tini@libero.it (P.T.); 9 Department of Precision Medicine, University of Campania “L. Vanvitelli”, 80138 Naples, Italy; amalia.luce@unicampania.it; 10 Biogem Scarl, Institute of Genetic Research, Laboratory of Precision and Molecular Oncology, 83031 Ariano Irpino, Avellino, Italy; 11 Medical and Translational Oncology Unit, Department of Experimental and Clinical Medicine, Magna Graecia University, 88100 Catanzaro, Italy (MOU-CZ); daniele.caracciolo@unicz.it (D.C.); tassone@unicz.it (P.T.); tagliaferri@unicz.it (P.T.); 12 Sbarro Institute for Cancer Research and Molecular Medicine and Center of Biotechnology, College of Science and Technology, Temple University, Philadelphia, PA 19122, USA; luciano.mutti@hotmail.it (L.M.); luigipirtoli@gmail.com (L.P.); giordano@temple.edu (A.G.); 13 Department of Medical Biotechnology, University of Siena, 53100 Siena, Italy

**Keywords:** cancer immunotherapy, PD-1/PD-L1 blockade, irAEs, immune-related pneumonitis, HLA-profile

## Abstract

Tumor-infiltrating T cell rescue by programmed cell death receptor-1 (PD-1)/PD-1 ligand-1 (PD-L1) immune checkpoint blockade is a recommended treatment for malignant diseases, including metastatic non-small-cell lung cancer (mNSCLC), malignant melanoma (MM), head and neck, kidney, and urothelial cancer. Monoclonal antibodies (mAbs) against either PD-1 or PD-L1 are active agents for these patients; however, their use may be complicated by unpredictable immune-related adverse events (irAEs), including immune-related pneumonitis (IRP). We carried out a retrospective multi-institutional statistical analysis to investigate clinical and biological parameters correlated with IRP rate on a cohort of 256 patients who received real-world treatment with PD-1/PD-L1 blocking mAbs. An independent radiological review board detected IRP in 29 patients. We did not find statistical IRP rate correlation with gender, tumor type, specific PD-1 or PD-L1 blocking mAbs, radiation therapy, inflammatory profile, or different irAEs. A higher IRP risk was detected only in mNSCLC patients who received metronomic chemotherapy +/− bevacizumab compared with other treatments prior PD-1/PD-L1 blockade. Moreover, we detected a strong correlation among the IRP rate and germinal expression of HLA-B*35 and DRB1*11, alleles associated to autoimmune diseases. Our findings may have relevant implications in predicting the IRP rate in mNSCLC patients receiving PD-1/PD-L1 blockade and need to be validated on a larger patient series.

## 1. Introduction

Immune checkpoint blockade with monoclonal antibodies (mAbs) to programmed cell death receptor-1 (PD-1) (nivolumab and pembrolizumab) and PD-1 ligand-1 (PD-L1) (atezolizumab, avelumab, and durvalumab) alone or in combination with chemotherapy, radiotherapy, or bevacizumab is an expanding treatment strategy for metastatic non-small-cell lung cancer (mNSCLC), malignant melanoma, head and neck cancer, kidney and urological cancer, and other common malignancies. These innovative immune-oncological treatments are leading to positive results in terms of clinical benefit and survival in these patients worldwide [1,2,3]; however, this therapeutic approach can be complicated by a number of immune-mediated adverse events (irAEs) that are mostly unpredictable, and require intensive medical intervention and sometimes lead to real-life-threatening situations. The occurrence of irAEs may have additional detrimental consequences derived from the discontinuation of an ongoing immune-oncological therapy in patients with no active alternative treatments [4,5,6]. Therefore, it is conceivable that the research of reliable biomarkers predictive of irAEs is eagerly awaited and encouraged worldwide. 

Immune checkpoint inhibitor-related pneumonitis (IRP) is an immune-related sub-acute disease that occurs in 4–5% of patients receiving anti-CTLA-4 and PD-1/PD-L1 immune checkpoint inhibitors (ICIs). This adverse event is almost unpredictable and usually occurs two/three months after the beginning of the treatment. It shows a higher frequency in patients who receive combination therapies compared to single agent-based therapy and may be severe (grade 3-4) in 15–20% of the cases [7,8].

Patients with NSCLC also show a higher frequency of IRP compared to malignant melanoma and renal cell carcinoma. Smocking habitus, previous use of taxanes, gemcitabines, tyrosine kinase inhibitors (TKIs), and radiotherapy have also been associated with a higher risk of IRP [9]. IRP pathogenesis seems to be related to alveolar damage as well as sarcoid-like granulomatosis reaction and interstitial fibrosis. In these patients, broncho-alveolar-lavages reveal a significant increase in lymphocytes (55–60%) with an elevated CD4+/CD8+ T cell ratio [8,10]. IRP onset is usually characterized by non-productive cough, tachycardia, asthenia, and dyspnea not associated with fever. Symptoms may progressively rise to hypoxia and cyanosis leading to respiratory failure [11].

It has been shown that pneumonitis may be observed by computed tomography (CT) scan weeks before it becomes clinically evident and often may remain asymptomatic [7,12]. CT scan may record not univocal pictures concerning IRP and, in most cases, it is described as aseptic organizing pneumonia, with ground glass or consolidative opacities with peribronchial and peripheral distribution. It is often associated with interstitial reaction and reticular opacities in the lower lung camps [7,9,12].

IRP has also been reported as a hypersensitivity disease with acute interstitial pneumonia and respiratory distress syndrome or sarcoid-like pulmonary changes with ilar lymph-node involvement that may mimic disease progression [12,13].

However, few data are available concerning the pathogenesis of adverse events associated with this treatment, even though a clear involvement of the immune-mediated T cell-response and T cell associated inflammatory response has been hypothesized. In this context, we carried out a multicenter retrospective analysis aimed to evaluate the frequency of IRP in our series and identify potential markers able to predict its occurrence in 256 cancer patients receiving PD-1/PD-L1 blockade for their specific disease. In particular, the risk of IRP was correlated with several clinical and biological markers and the germinal expression of class I and class II human leukocyte antigen (HLA) alleles.

## 2. Materials and Methods

### 2.1. Patients Sample, Treatment, and Monitoring

This work is part of a retrospective real-world evidence (RWE) multi-institutional study including 256 cancer patients who had received therapy with anti-PD-1 (nivolumab or pembrolizumab) or anti-PD-L1 (atezolizumab) for their specific disease at the OU-RC, MOU-CZ, and ROU-SI between September 2015 and January 2020. Patients’ features and/or other eventual treatments prior to immune checkpoint blockade are reported in Table 1 and Table 2.

All patients gave an informed consent for the anonymous use of their clinical data for the research aim. All procedures were undertaken in compliance with the ethical statements of the Helsinki Declaration (1964, amended most recently in 2008) of the World Medical Association and respect of their privacy. All patients received PD-1/PD-L1 blockade as recommended by international guidelines and regulative agencies following the standard procedures of administration for each specific drug. All patients according to their specific condition received: a) Intravenous pembrolizumab (200 mg every three weeks) (29 patients) or b) nivolumab (3 mg/kg every two weeks) (189 patients), or c) atezolizumab (1200 mg every three weeks) (38 patients) until disease progression or severe adverse events. 

All patients were fit for treatment (Eastern Cooperative Oncology Group (ECOG) performance status ≤ 1). A complete physical examination report, histological sampling, hematologic, biochemical, immune-biological, radiological, and instrumental monitoring were available at baseline. Clinical history, physical examination, and record of adverse events were evaluated prior to each drug infusion. A CT scan was performed every 3 months or in any case of suspected progressive disease (PD) and evaluated according to the immune Response Evaluation Criteria in Solid Tumors (iRECIST) [14].

Patients were monitored for blood cell counts, and biochemistry prior to each treatment course and were also monitored for their adrenal hormone profile, ACTH, TSH, thyroid hormones, anti-thyroid autoantibodies (aAbs), extractable nuclear antigen antibodies (ENA), anti-nucleus antibodies (ANA), anti-smooth cells antibodies (ASMA), and c/p- anti-neutrophil cytoplasmic antibodies (ANCA) each month from the beginning of treatment.

This study also included patients who had been voluntary screened for A, B, C, and DRB1 HLA typing in the Tissue Typing Unit at the Grand Metropolitan Hospital in Reggio Calabria by performing low-medium-resolution reverse SSO DNA typing assays (One-Lambda Luminex Technology LABScan™ 100, HLA Fusion™ Software, One Lambda Inc., Thermo Fisher Scientific, Los Angeles, CA, USA) on genomic DNA extracted by whole blood or peripheral blood mononuclear cells (PBMCs) according to the kit manufacturers. The HLA allele frequency of the studied loci A, B, C, and DRB1 in our patients’ cohorts was obtained by comparing the results with our database bank including 3500 healthy bone marrow donors of the Calabrian National Registry.

CT scan images of these patients were blindly evaluated by a radiology review board composed of AS (OU-RC) and MAM (RU-SI) for the research of radiological signs of immune-related pneumonitis at baseline, after 3 and 6 months of treatment, or when they showed pneumonitis related to clinical signs. 

### 2.2. Statistical Analysis

Time to events were analyzed with the Kaplan–Meier method and survival analysis was performed by the log-rank test. Median survival and 95% confidence intervals were reported. Median follow-up was estimated with the reverse method. Hazard ratios (HRs) and their 95% confidence intervals were estimated through the Cox regression proportional model; in the multivariate approach, a forward stepwise procedure was used and the enter and remove limit set to 0.05 and 0.10, respectively. Association between irAEs and clinical and biological parameters was assessed by the chi-square test. Statistics were performed by the SPSS software 23.0 (International Business Machines Corp., New York, NY, USA).

## 3. Results

### 3.1. Patients’ Features

Our analysis included a cohort of 256 patients (mean age 64.0 ± SD 10.4 years), with 194 (75.8%) males, (mean age 64.9 ± SD 9.8 years) and 62 (24.2%) females (mean age 61.3 ± SD 11.5 years). Sixty-two patients (24.2%) presented with a squamous lung cancer histology and 127 (49.6%) with a non-squamous lung cancer histology and the remaining 67 (26.2%) with other histology different from lung cancer. 

In total, 189 patients (including all histologies) received nivolumab as a therapy for metastatic disease (of these 138 were mNSCLC patients), 29 patients were treated with pembrolizumab (of these 20 were mNSCLC patients), and 38 patients with atezolizumab (of these 31 were mNSCLC patients). In total, 101 (56.4%) mNSCLC patients had received a frontline standard doublet chemotherapy and 67 (37.4%) a platinum-based metronomic chemotherapy +/− bevacizumab therapy. Ninety-two (48.7%) of the mNSCLC patients had also received palliative radiotherapy (25–30 Gy) on symptomatic single lesions (mediastinum, nodes, soft tissue, bone, or brain) and 30 (15.9%) patients received prior TKI (erlotinib) before ICI treatment (Table 1 and Table 2).

### 3.2. Predictive Values of Clinical Parameters

PD-1/PD-L1 immune checkpoint blockade was generally well-tolerated and there was no treatment-related death. IrAEs were observed in 68 (26.6%) patients including also other histology and mainly consisted in grade 1-2 cutaneous rash, polyarthritis, and thyroiditis, generally occurring after 3–4 treatment courses not requiring medication. Among them, more severe irAEs were recorded in 18 patients (7.03%) who showed clinical, laboratory, and radiological signs of hypophysitis (1 case), poly-mucositis (1 case), adrenalitis (2 cases), and symptomatic IRPs (14 cases; 5.4%, all recorded in NSCLC patients) (Table 2). The latter patients presented respiratory symptoms, requiring medical treatment and 11 of them required permanent discontinuation of the immune-oncological treatment.

### 3.3. Immune-Related Pneumonitis by a Centralized Radiological Board Review

**Figure 1:**
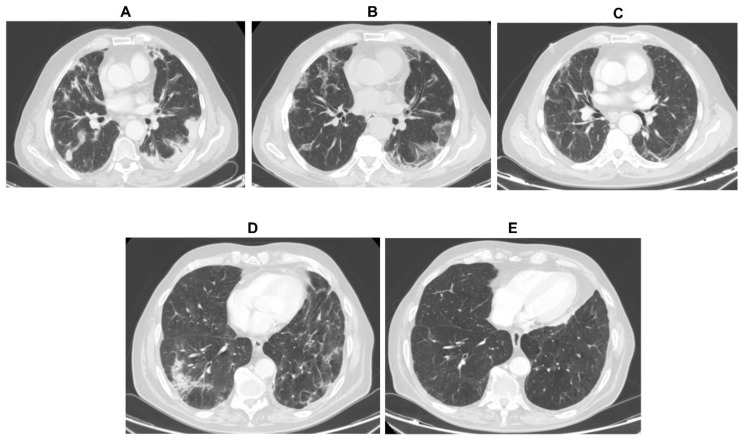
Computed tomography (CT) scan imaging. Panel (**A**–**C**): 80-year-old male with metastatic non-small-cell lung cancer (mNSCLC) showing a classical cryptogenic organized pneumonia pattern. (**A**) Axial chest CT image obtained after 10 months of treatment with nivolumab showing multiple consolidations involving both lungs. (**B**) Axial follow-up CT-image obtained 1 month later showing an improvement of pneumonitis and the presence of ground-glass opacities. (**C**) Axial follow-up CT image obtained 11 months later showing pneumonitis resolution and the presence of bilateral fibrotic alteration. Panel (**D**–**E**): 70-six-year-old male with mNSCLC presenting a not otherwise specified pattern of pneumonitis. (**D**) Axial chest CT image obtained 2 months after the beginning of the treatment with nivolumab showing multiple consolidations, interlobular septal thickening, subpleural reticulations. (**E**) Axial chest CT image obtained 8 months later showing a complete resolution of the signs.

An independent imaging review board analysis was carried out on the CT images of all the patients included in the study. The CT scans were performed at baseline and during treatment as a standard radiological monitoring. Five distinct IRP signs were searched according to the literature classification: (i) Cryptogenic organized pneumonia-like, (ii) ground glass opacities, (iii) interstitial pneumonitis, (iv) hypersensitivity, and (v) pneumonitis not otherwise specified [8]. As a whole, this centralized radiological analysis determined IRP in 29 patients (11.3%), including the 14 (5.4%) known cases who reported clear respiratory symptoms and an additional 15 cases (5.9%) that were completely asymptomatic (among them 7 patients had non-squamous non-small-cell lung cancer (nsqNSCLC), 3 squamous non-small-cell lung cancer (sqNSCLC), 2 melanoma, and 3 other histology) (Figure 1 and Table 2).

### 3.4. Laboratory Analysis and HLA Typing

Patients who received the immunological treatment in our series were monitored for changes in blood cells, inflammatory markers, and aAbs. They reported a significant increase in ANA, ENA, and ASMA as described in previous studies by our group [15]. In total, 180 patients were voluntarily evaluated for germline expression of class I HLA A, B, C loci, and DRB1 for class II. Our research of the two most frequent alleles for each locus detected A*02 (94 patients) and A*01 (54 patients); B*18 (47 patients) and B*35 (44 patients); C*07 (94 patients) and C*04 (46 patients); and DRB1*11 (88 patients) and DRB1*03 (37 patients). In our analysis, we also included allele HLA-B*51 (39 patients) for its known correlation with common autoimmune diseases [16,17]. Allele homozygosis for the loci A, B, C, and DRB1 were, finally, recorded in 23, 16, 40, and 26 cases, respectively. These results were in line with the HLA frequency reported in the healthy bone marrow donor database in the Calabrian Regional Registry, representative of the healthy population in this geographic area.

### 3.5. Statistical Correlation

**Figure 2:**
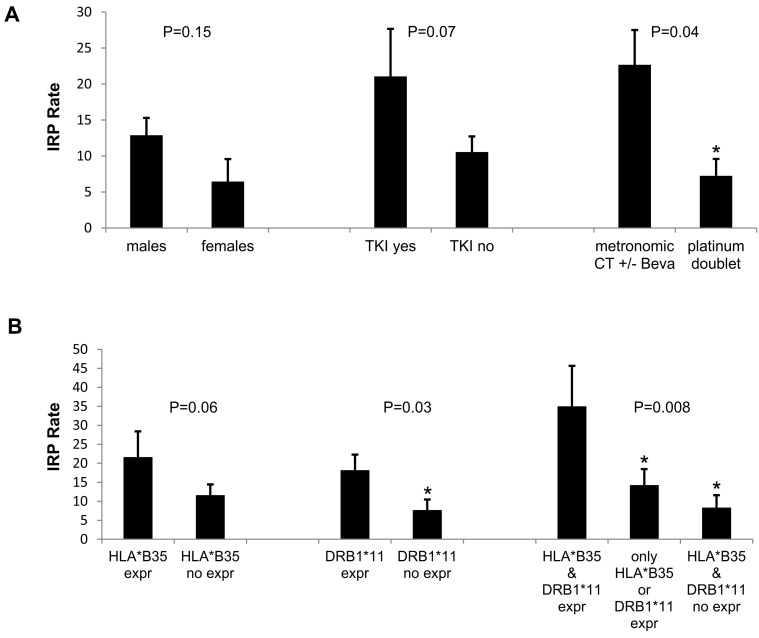
Rate of immune checkpoint inhibitor-related pneumonitis (IRP) in metastatic non-small-cell lung cancer (mNSCLC) patients. (**A**) Histograms relative to the rate of IRP in patients stratified for gender (considering the whole population including also histology different from NSCLC), tyrosine kinase inhibitor (TKI) therapy, and metronomic chemotherapy with fractioned cisplatin and oral etoposide (mPE)/metronomic chemotherapy with fractioned cisplatin and oral etoposide + bevacizumab (mPEBev) regimen prior to PD-1/PD-L1 immune checkpoint inhibitors. (**B**) Histograms relative to the rate of IRP in all of the evaluated patients compared in terms of positive expression of the HLA-B*35 allele alone, DRB1*11 allele alone, and both alleles.

Our analysis failed to correlate the percentage of the IRP rate with tumor type, PD-1 or PD-L1 blocking mAbs administration, prior radiation therapy, occurrence of other irAEs, or inflammatory markers, as well as aAbs’ rise (data not shown; Supplementary Materials Table S1). The IRP rate was calculated considering the % of patients who developed IRP after administration of ICIs. On the other hand, the IRP rate of all the population including all histology showed a trend, without reaching statistical significance, to higher risk in males than females (25 out 194 males, 12.9%; and 4 out 62 females, 6.5%; *P* = 0.15). Moreover, the rate of IRP was significantly increased in mNSCLC patients who had received frontline metronomic chemotherapy +/− bevacizumab (*P* = 0.04) prior to PD-1/PD-L1 blockade while no correlation was found with previous treatment with TKIs (Figure 2A). Finally, the IRP rate was correlated with the expression of HLA-B*35 (27.6% vs. 11.6%; *P* = 0.06) and DRB1*11 (21.0% vs. 7.6%; *P* = 0.03) alleles. The highest rate of IRP was detected in those patients co-expressing B*35 and DRB1*11 (*P* = 0.008) (Figure 2B).

No IRP rate correlation was conversely detected with class I HLA-A or C alleles as well as with the status of heterozygosis of class I HLA, A, B, and C and DRB-1.

**Figure 3:**
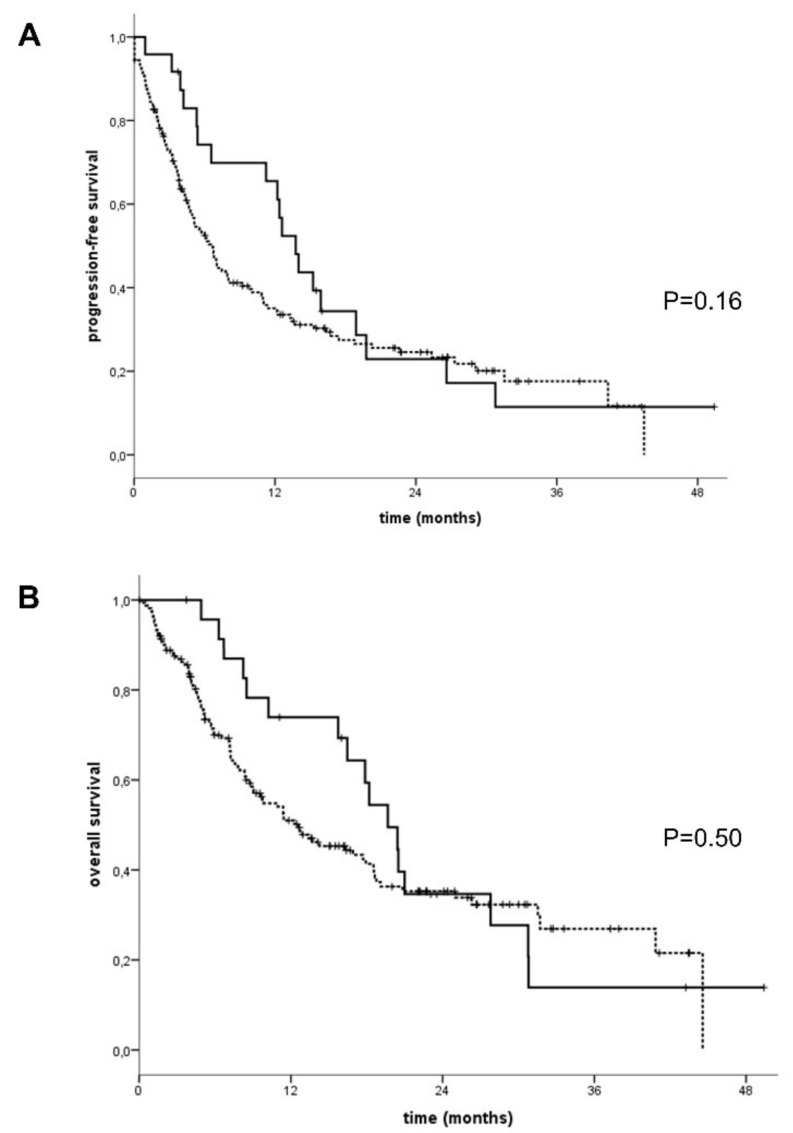
Kaplan–Meyer curves. (**A**) Median progression-free survival (PFS) and (**B**) Overall survival (OS) of metastatic non-small-cell lung cancer (mNSCLC) patients under treatment with PD-1/PD-L1 immune checkpoint inhibitors who showed (continuous line) or not (dashed line) radiological signs of IRP. Curves show a not statistically significant trend of longer PFS and OS in patients who present IRP compared to the others, respectively (PFS; 13.8 (95% CI: 11.2–16.3) vs. 6.7 (95% CI: 5.3–8.1) months, *P* = 0.16. OS; 19.7 (95% CI: 16.0–23.3) vs. 12.6 (95% CI: 7.8–17.4) months; *P* = 0.50).

Considering that other irAEs showed a tight correlation with patients’ outcome, we also compared the IRP frequency with survival in mNSCLC patients. Indeed, we found a trend to a prolonged survival that, however, did not reach statistical significance. Particularly, patients with IRP compared to the others, showed a trend to a better progression-free survival (PFS) 12.4 (95% CI: 9.3–15.5) vs. 4.9 (95% CI: 3.6–6.2) months; *P* = 0.16] and overall survival (OS) 18.2 (95% CI: 13.4–23.0) vs. 11.4 (95% CI: 8.5–14.3) months; *P* = 0.50) (Figure 3).

In our series, either HLA-B*35 or DRB1*11 allele expression were not correlated to better PFS and OS of NSCLC patients subjected to immune checkpoint blockade (data not shown, Supplementary Materials Figure S1).

## 4. Discussion

This retrospective study was carried out in patients affected by mNSCLC and other cancers who received a real-world immunotherapy with mAbs to PD-1 (nivolumab and pembrolizumab) or PD-L1 (atezolizumab). The results of this study fulfilled the primary endpoint to identify predictive and biological markers of the IRP rate. Firstly, our centralized review, which aimed to identify radiological signs of IRP independently of the symptoms, revealed a twice higher rate of this adverse event compared to that reported in the literature (11.3% vs. 4%) [7,8,9]. This finding could be explained by a specific radiological study that allowed the cases with asymptomatic pneumonitis to be recognized, suggesting that the IRP frequency could be largely underestimated. In fact, in our series, almost more than half of the patients (15 out of 29 cases) had radiological evidence of pneumonitis without clinical signs that completely reversed upon appropriate steroid medication, allowing the patients to continue the immune checkpoint blockade therapy after temporary discontinuation. On the other hand, 11 out of 14 patients with respiratory symptoms were partially refractory to the steroid treatment, thus requiring permanent discontinuation of ICIs and when possible a change of treatment. However, no IRP-related death was recorded in our series. Our statistical analysis failed to demonstrate a clear correlation with histology, gender, radiation therapy, and different ICIs even though a trend to a higher IRP frequency was observed in male patients with NSCLC. Differently, we found a greater rate of IRP in mNSCLC patients who had received metronomic chemotherapy with fractioned cisplatin and oral etoposide -/+ bevacizumab (mPE/mPEBev regimen) prior to starting the immune checkpoint blockade therapy. The latter finding was not surprising considering that this group of NSCLC patients showed the highest frequency of self-limiting irAEs and the best outcome in previous studies [15]. The mPE/mPEBev is a very active treatment regimen designed on translational bases that showed immune-modulating effects resulting in a peripheral decline of regulatory T cells (Tregs) and an increase in activated DCs and central memory T cells (Tcm) in mNSCLC patients [18,19,20,21,22,23]. On the other hand, another platinum-based chemotherapy agent, such as oxaliplatin, in combination with gemcitabine, leucovorin and 5-fluorouracil (GOLF regimen) was already known to induce the generation of CTL precursors with enhanced antitumor activity on colon cancer cells in vitro as a result of an extensive delivery of antigenic material from tumor cells [24,25,26].

In this context, our hypothesis that IRP could be related to patients’ HLA genotype was achieved. As shown in previous reports, class I and II HLA allele profiling have been correlated to either treatment response or survival in cancer patients receiving immune checkpoint blockade [27,28,29]. In fact, HLA complex plays a critical role in both the efficiency and modulation of the T cell-mediated immune response, performing the specific function of presenting tumor antigen-derived peptides to specific T cell receptors on effector T cells [26,27,28]. The epitope number as well as the peptide affinity for HLA molecules is haplotype specific, thus the same antigen may have different immunogenicity in individuals with different HLA profiles. In this light, class I HLA A B and C have the specific function of presenting peptide epitopes to cytotoxic T cells either on target cells or antigen-presenting cells in the lymph nodes. On the other hand, class II HLA-DR, DP, and DQ, expressed on myeloid cells and antigen presenting cells (APCs) are involved in presenting antigen-derived peptides to regulatory (tregs) or T helper lymphocytes. Therefore, they are able to repress or enhance specific T cell-mediated or antibody response to specific antigens. In this light, it has been largely demonstrated that some class I as well as class II HLA have been tightly correlated to specific inflammatory autoimmune diseases and viral infection susceptibility [30,31,32,33,34,35].

In our series, IRP was correlated to the expression of HLA-B*35 and/or DRB1*11 alleles. B*35 is a frequent class I HLA allele expressed on the surface of both target cells and activated APCs able to present antigen-derived peptides to cytotoxic T cells and relative precursors. The product of the HLA-B*35 gene has been associated, in patients with autoimmune disease, to endoplasmic reticulum stress (ERS), a process that promotes both the inflammatory status and the expansion of inflammation-associated cell lineages. For this purpose, it has been associated to a high risk of *Chlamydia pneumoniae*-associated pneumonitis in patients with chronic diseases and severe primary pulmonary hypertension in patients with scleroderma and nephritis associated to hyper-leukocytosis disease [36,37,38]. Similarly, B*35 expression has also been associated to high-risk juvenile idiopathic arthritis [39].

On the other hand, DRB1*11 represents the alpha unit of the most frequent class II HLA-DRB1 alleles in Caucasians. It is expressed on APCs and it can present antigen-derived peptides to CD4+ T cells, affecting the dynamic balance between cytotoxic T cell (Th1) and B cell/antibody (Th2) response. HLA-DRB1*11 expression has been associated to the rate of systemic sclerosis and promotes the induction of anti-DNA topoisomerase I Abs [40]. Moreover, its expression may impair the T cell cytotoxic response to specific viruses, leading to hepatitis B and C viruses’ persistence and HPV-associated cervical cancer [41,42].

On these bases, the proinflammatory and ERS induced by the HLA-B*35 allele as well as the Th2-promoting ability of DRB1*11 might explain the increased rate of IRPs in cancer patients receiving PD-1/PD-L1 immune checkpoint inhibitors. The pathogenesis of IRP is not clear, and it is not demonstrated if there is an inappropriate cell-mediated response to sequestered alveolar antigens or latent pathogens. However, several pathological studies show that IRP is correlated with a delayed alveolar inflammatory reaction with high infiltration of CD4+ T cells, which are not directly influenced by mAbs to PD-1/PD-L1. In this light, previous treatments with immune-modulating drug combinations, such as taxanes, mPE +/- bevacizumab, or TKI, may prepare the alveolar tissue for immune-mediated inflammatory attack. It is of interest the finding that IRP mainly occurs in patients responsive to immune checkpoint inhibitors with NSCLC where T cell-mediated cytotoxic damage of alveolar cells is enhanced by the concomitant presence of tumor cells in the organ.

In line with the results of other immunological studies, the occurrence of irAEs in these patients is consequential to the cross-priming of antigens released from the tumor site in an immunological context of CTL-mediated immune-rejection, whose cross-priming may be facilitated and redirected to the B cell compartment by the germline expression of specific and multiple class II HLA alleles [4,43,44].

If these data should be confirmed on a large series of patients, it will be possible to identify a risk population that requires careful monitoring when subjected to ICIs’ administration alone or in multidrug combination regimens.

Recent findings concerning the combination of PD-1/PD-L1 blockers with front-line chemotherapy in advanced NSCLC patients compared to the same chemotherapy doublet alone were partially sorrowed by the occurrence of a high risk of early deaths (within 60 days of treatment) due to severe immunological disorders [45,46]. On these bases, it is possible to speculate that immune system overstimulation combining danger signals and massive antigen release consequent to secondary cytotoxic agents (anticancer drugs/radiotherapy/anti-angiogenic agents) might be detrimental in patients who already show an inappropriate immune response [45,46].

## 5. Conclusions

Based upon the results of the present study, we believe that an accurate definition of tumor immunogenicity prior to starting therapy as well as germline class I and II HLA allele characterization have to be validated on a large-scale patient population on real-world evidence in order to define both the responsiveness and risk of lethal irAEs. The prediction of irAEs is becoming of pivotal importance considering both the possibility to combine different ICIs alone or with other anticancer agents and the consequent need to prevent a dangerous cell-mediated inflammatory response. Our findings may also of be interest considering emerging Severe Acute Respiratory Syndrome-Coronavirus-2 (SARS-CoV-2)-related pneumonitis and their potential correlation with germline class I and II HLA alleles, suggesting a common pathogenesis of the two conditions.

## Figures and Tables

**Table 1:** Clinical characteristics of patients.

Hystology	Number of Patients	Sex	RT	TKI	First-Line Treatment Prior ICIs
SqNSCLC	62	M: 55; F: 7	Y: 27; N: 35	Y: 10; N: 52	None: 2 Standard doublet: 41 mPE/mPEBev: 18
NsqNSCLC	127	M: 97; F: 30	Y: 65; N: 62	Y: 20; N: 105	None: 16 Standard doublet: 60 mPE/mPEBev: 49
Other	67	M: 42; F: 25	Y: 16; N: 38; NA: 13	NA	NA

SqNSCLC: squamous non-small-cell lung cancer, NsqNSCLC: non-squamous non-small-cell lung cancer, Other: melanoma, head and neck cancer, kidney, urothelial, gastro-enteric, and breast cancer, RT: radiotherapy, TKI: tyrosine kinase inhibitor, First-line treatment prior ICIs: first-line treatment prior immune checkpoint inhibitors, Y: yes, N: no, mPE: metronomic chemotherapy with fractioned cisplatin and oral etoposide, mPEBev: metronomic chemotherapy with fractioned cisplatin and oral etoposide + bevacizumab, NA: not applicable.

**Table 2:** Immune-related pneumonitis (IRP) and immuno-related adverse events (irAEs).

Hystology	Atezo	IRP	Pembro	IRP	Nivo	IRP	Overall IRP	Overall irAEs
SqNSCLC	10	S: 0 NS: 1	1	S: 0 NS: 0	51	S: 3 NS: 2	S: 3 (4.8%) NS: 3 (4.8%) S+NS: 6 (9.7%)	18 (29%)
NsqNSCLC	21	S: 2 NS: 0	19	S: 0 NS:2	87	S: 9 NS: 5	S: 11 (8.7%) NS: 7 (5.5%) S+NS: 18 (14.2%)	45 (35.4%)
Melanoma	0	S: 0 NS: 0	3	S: 0 NS: 0	16	S: 0 NS: 2	S: 0 (−) NS: 2 (10.5%)	2 (10.5%)
Miscellaneous	7	S: 0 NS: 0	6	S: 0 NS: 0	35	S: 0 NS:3	S: 0 (−) NS: 3 (7.5%)	3 (7.5%)

SqNSCLC: squamous non-small-cell lung cancer, NsqNSCLC: non-squamous non-small-cell lung cancer, Atezo: number of patients who received atezolizumab, Pembro: number of patients who received pembrolizumab, Nivo: number of patients who received nivolumab, irAEs: immuno-related adverse events, IRP: immune-related pneumonitis, S: symptomatic IRP, NS: non-symptomatic IRP, Miscellaneous: head and neck cancer, kidney, urothelial, gastro-enteric, and breast cancer.

## Supplementary Materials

The following are available online at https://www.mdpi.com/2073-4409/9/9/1964/s1, Figure S1: Kaplan Meyer curves. Table S1: IRP correlation with blood inflammatory markers.
