# Supplementary material for: HLA Expression Correlates to the Risk of Immune Checkpoint Inhibitor-Induced Pneumonitis
Source: Cells. 2020 Aug 25;9(9):1964. doi: 10.3390/cells9091964 (PMC7564884; doi:10.3390/cells9091964)
Supplement: Supplementary file 1 [file cells-09-01964-s001.zip › Table S1.pdf]

Table S1. IRP correlation with blood inflammatory markers. AAbs rise as well as homozygosis status in germline HLA A, B, C and DRB1 alleles did not correlate with IRP rate.

	IRP	N	Avg	Std Dev	P-value
**Neutr**	No	137	5.43	3.14	0.16
	Yes	26	4.50	2.49	
**Lymph**	No	137	1.79	1.54	0.27
	Yes	25	1.44	0.51	
**NLR**	No	133	4.38	4.96	0.38
	Yes	25	3.49	2.26	
**MON**	No	137	0.75	0.39	0.32
	Yes	26	0.66	0.30	
**EOS**	No	137	0.13	0.15	0.94
	Yes	26	0.14	0.14	
**CRP**	No	106	25.66	42.21	0.16
	Yes	22	12.60	19.75	
**ESR**	No	94	46.02	34.12	0.98
	Yes	21	46.19	25.42	
**LDH**	No	126	462.95	277.66	0.09
	Yes	24	362.04	209.25	

Neutr: neutrophils, Lymph: lymphocytes, NLR: neutrophil to lymphocyte ratio, MON: monocytes, EOS: eosinophils, CRP: C-reactive Protein, ESR: erythrocyte sedimentation rate, LDH: lactate dehydrogenase, IRP: immune-related pneumonitis, N: number of patients enrolled for the analysis, Avg: average values, Std Dev: standard deviation of the values.
