# Supplementary figures and images for: HLA Expression Correlates to the Risk of Immune Checkpoint Inhibitor-Induced Pneumonitis
Source: Cells. 2020 Aug 25;9(9):1964. doi: 10.3390/cells9091964 (PMC7564884; doi:10.3390/cells9091964)

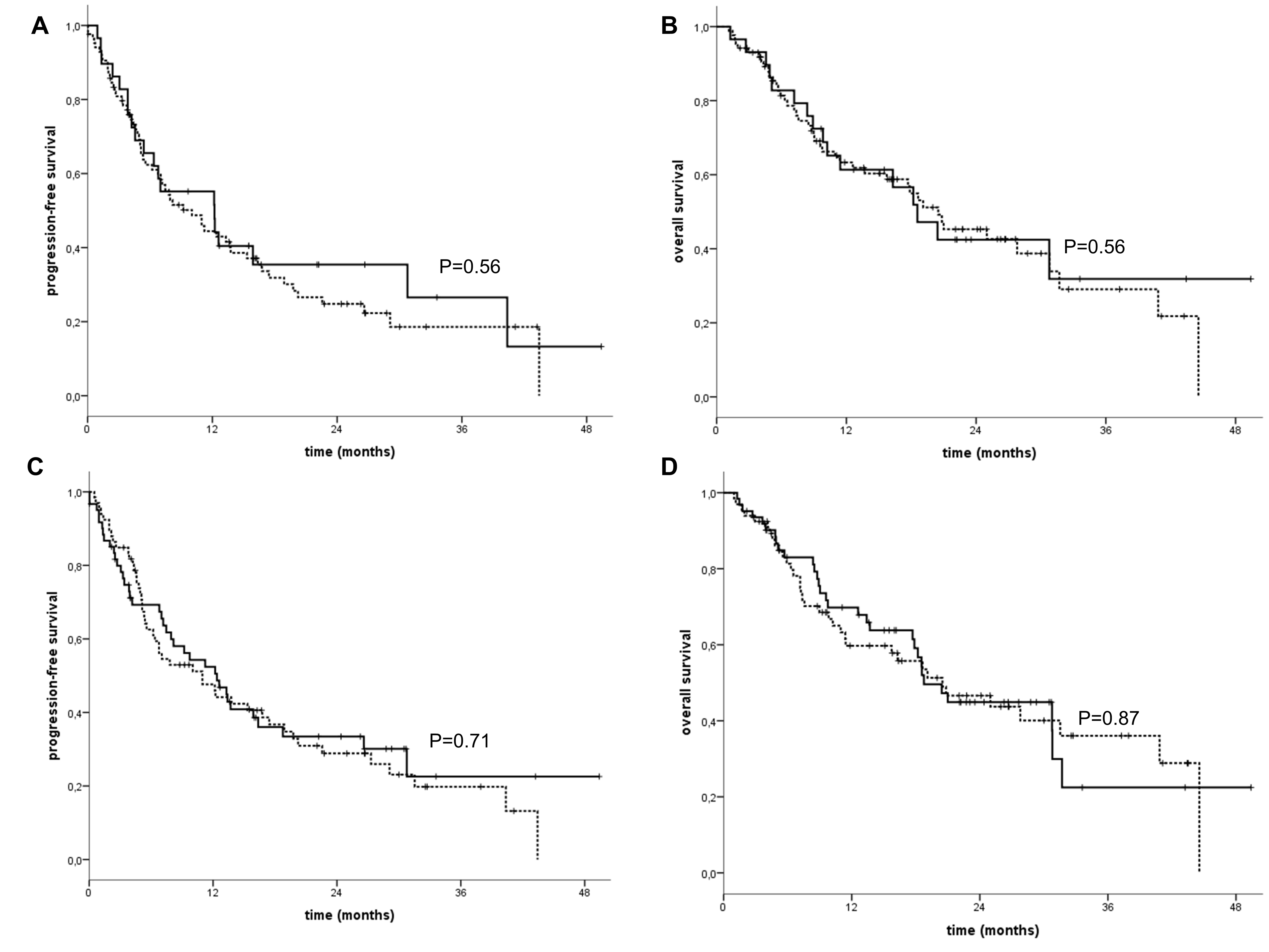

Supplement: Supplementary file 1 [file cells-09-01964-s001.zip › FIGURE S1.tif]
